# Uncovering the Reasons Behind Maternal Care Dropout in Bangladesh: Cross-Sectional Study

**DOI:** 10.2196/85875

**Published:** 2026-04-01

**Authors:** Syeda Saima Alam, Plabon Sarkar, M A Rifat, Sumaiya Jahan, Rokibul Islam, Israt Jahan, Sanjib Saha

**Affiliations:** 1 Noakhali Science and Technology University, Noakhali, Bangladesh; 2 University of Dhaka, Dhaka, Bangladesh; 3 BRAC University, Dhaka, Bangladesh; 4 Lund University, BMC, Sölvegatan 19, Lund, 22362, Sweden, 46 72 467 48 80

**Keywords:** maternal dropout, continuum of care, antenatal care, skilled birth attendants, postnatal care, demographic and health survey, Bangladesh

## Abstract

**Background:**

Utilization of the maternal continuum of care (CoC)—comprising adequate antenatal care (ANC), skilled birth attendance, and postnatal care (PNC)—is critical for improving maternal and child health outcomes. However, dropout from the CoC remains substantial in Bangladesh, with women discontinuing services at different stages of pregnancy, delivery, and postpartum care.

**Objective:**

This study aimed to quantify maternal dropout at each stage of the CoC and identify socioeconomic and demographic factors associated with discontinuity, comparing two nationally representative survey rounds.

**Methods:**

Data were drawn from the Bangladesh Demographic and Health Surveys (BDHS) 2017‐2018 and 2022. Women aged 15 to 49 years with a live birth in the preceding 2 to 3 years were included. Completion of full CoC was defined as receiving at least 4 ANC visits, delivering with a skilled birth attendant, and obtaining at least 1 PNC contact within 48 hours of delivery. Predisposing (age, education, parity, religion, and division), enabling (wealth index, media exposure, health care access, and residence), and need factors (terminated pregnancy and desired pregnancy status) were identified using the Andersen Behavioral Model. Survey-weighted multivariable logistic regression models were fitted for each CoC component and overall CoC completion, with interaction terms to assess whether associations differed between survey rounds.

**Results:**

Among 8424 mothers, 27.9% (n=2350) failed to complete all components of the maternal CoC. Dropout was highest at the ANC stage (n=4962, 55.7%), followed by PNC (n=3976, 47.2%) and skilled birth attendant–assisted delivery (n=3378, 40.1%). Between survey rounds, overall CoC dropout decreased significantly from 31.9% (BDHS 2017‐2018) to 22.4% (BDHS 2022), reflecting modest improvements in service continuity. Factors significantly associated with higher odds of CoC dropout included lower maternal education (adjusted odds ratio [AOR] 2.70, 95% CI 1.94‐3.77; *P*<.001), higher parity (AOR 2.73, 95% CI 2.12‐3.50; *P*<.001), lower wealth quintiles (AOR 4.04, 95% CI 3.02‐5.41; *P*<.001), and rural residence (AOR 1.40, 95% CI 1.18‐1.67; *P*<.001). Protective factors included older maternal age at delivery (AOR 0.56, 95% CI 0.42‐0.74; *P*<.001) and history of ever-terminated pregnancy (AOR 0.74, 95% CI 0.63‐0.86; *P*<.001). Significant temporal interactions (all *P*<.05) indicated that the strength of associations for education, parity, religion, wealth, media exposure, health care access barriers, residence, and pregnancy desire differed between survey rounds, reflecting changing determinants of CoC engagement amid policy reforms and pandemic disruptions.

**Conclusions:**

Maternal, socioeconomic, and geographic factors are strongly associated with discontinuity along the maternal health care continuum in Bangladesh. Statistically significant temporal variations underscore the impact of evolving health policies and system disruptions on maternal service utilization patterns. Targeted, area-specific interventions addressing these determinants across all CoC components are essential to improve maternal health care retention and achieve better maternal and child health outcomes.

## Introduction

The maternal continuum of care (CoC) consists of 4 or more antenatal care (ANC) visits, childbirth by a skilled birth attendant (SBA), and a postnatal care (PNC) visit by skilled health care providers within 48 hours of delivery [1]. When a woman and child pair fulfills all 3 criteria, they are considered to have attained a complete CoC [1]. The CoC encompasses integrated service delivery for mothers and children, ensuring that women have access to quality care throughout pregnancy, childbirth, and the postpartum period [2]. It is critical for improved health outcomes, efficient and effective delivery of nutritional interventions, increased service utilization, and addressing underlying factors [3], with recent multicountry evidence underscoring that completion of the CoC remains suboptimal, particularly in the low- and middle-income countries [4].

The completion of CoC demonstrates a marked reduction in undernutrition indicators, for example, stunting, wasting, and underweight among children alongside a decline in maternal and child mortality rates [3,5]. Empirical research indicates that adequate continuity of care can avert half a million maternal fatalities, 4 million neonatal fatalities, and 6 million pediatric fatalities worldwide [6-8]. Consequently, withdrawal from the CoC signifies noncompliance with prescribed maternal care measures [9], and emerging findings from Bangladesh indicate that the use of the full maternal CoC is positively associated with optimal complementary feeding practices among children aged 6 to 23 months [10].

In Bangladesh, around 53% of pregnant women drop out from receiving 4 or more ANC visits, 47% of the childbirths are not supported by SBAs, and 48% of women do not receive PNC visits within 2 days after the delivery [11,12]. The dropouts of women and children from the CoC can severely impact maternal and child nutrition. For instance, lower ANC and PNC visits result in poor infant and young child feeding practices that increase the risk of malnutrition and impaired cognitive development, which may result in long-term adverse educational and economic outcomes for children [13]. In addition, insufficient ANC visits and nutrition counseling can lead to maternal malnutrition, such as anemia and pregnancy complications, while a lack of SBA during delivery and PNC visits might pose a risk to maternal mortality and poor reproductive health [13]. Considering the significance of the CoC components, they are being evaluated for consistency to safeguard the survival and welfare of both mother and newborn. This, in turn, may serve as a crucial strategy for achieving the Sustainable Development Goals’ target of a maternal mortality ratio under 70 per 100,000 live births and a neonatal mortality rate under 12 per 1000 live births by 2030 [6,13].

The CoC is essential for enhancing maternal and child nutrition outcomes, as interruptions in one service frequently cause disruptions in others. For example, data from Bangladesh indicate that women who cease ANC are more inclined to also abandon skilled birth attendance and PNC [13,14]. It is important to understand the many factors that lead to dropout in both individual and combined parts of the CoC to create complete and effective interventions. Key factors—such as maternal age, socioeconomic status, education level, place of residence, religion, parity, and occupation—significantly influence adherence to and gaps within the continuum, affecting the success of maternal and child nutrition initiatives [13,15]. To create targeted and comprehensive services that can remove barriers, make sure services continue, and ultimately improve the health and nutrition of mothers and children in Bangladesh, it is important to identify and analyze these factors in a strong way.

This study aims to comprehensively quantify and compare maternal dropouts at each stage of the CoC (ANC, delivery, and PNC) and from the overall CoC between Bangladesh Demographic and Health Surveys (BDHS) 2017-2018 and BDHS 2022, and to identify and contrast the socioeconomic and demographic factors associated with these discontinuities. It is hypothesized that the magnitude and pattern of maternal dropouts differ significantly between BDHS 2017‐2018 and BDHS 2022 across the stages of the CoC and for the overall CoC, and that the socioeconomic and demographic determinants of discontinuity at different stages of the CoC differ in strength and direction between the 2 survey rounds.

By exploring where and why women disengage from critical services and by comparing 2 distinct survey rounds, the research provides crucial insights into gaps that undermine maternal and child health outcomes. The findings from this study will help inform targeted strategies and policy interventions to reduce dropouts, improve service uptake, and ultimately contribute to achieving maternal and child health goals nationwide.

## Methods

### Study Design, Data Sources, and Sampling

This study used data from BDHS 2017‐2018 and BDHS 2022 to determine how CoC utilization and its covariates have changed over time. Both surveys are nationally representative, are cross-sectional in design, and employed 2-stage stratified random sampling. The overall response rate for both surveys was 98%. The detailed methods are outlined elsewhere [11,16].

A total of 8424 ever-married women aged 15 to 49 years who had a live birth within the last 3 and 2 years prior to the surveys, respectively, for BDHS 2017‐2018 and BDHS 2022, were retrieved. However, the study only analyzed samples with complete information regarding maternal utilization of CoC and coupled with respective covariates.

### Conceptual Model

**Figure 1.**
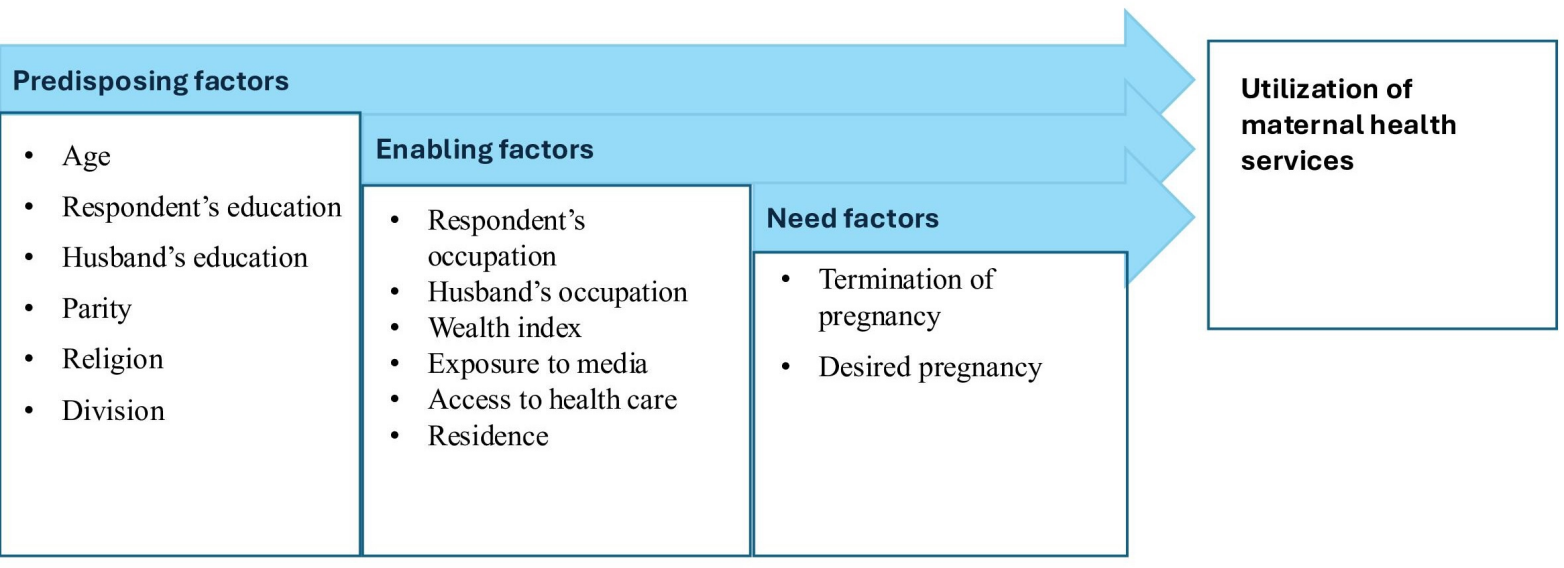
Theoretical framework based on the Andersen Health Care Utilization Model.

This study utilized the Andersen Behavioral Model of Health Care Services to systematically identify factors affecting the utilization of CoC services. The Andersen model is a widely used conceptual framework for understanding health care utilization, positing that service use is shaped by 3 key domains: predisposing, enabling, and need factors [17,18]. Predisposing factors encompass demographic and social attributes, including age, gender, education, and health beliefs, that affect an individual’s propensity to pursue care. This study identified maternal age, respondent’s education, husband’s education, parity, religion, and division as predisposing factors. Maternal age and parity are recognized factors influencing obstetric risk and health-seeking behavior, whereas maternal education affects health literacy, awareness of maternal health risks, and autonomy in decision-making. The husband’s education is a significant predisposing factor, as it indicates social status, influences household health beliefs, and affects decision-making authority concerning maternal health care utilization [19]. Enabling factors include the means and resources that make it easier or harder to get services, such as community resources, household wealth, and health care availability [20]. This study identified the respondent’s occupation, the husband’s occupation, wealth index, media exposure, health care access, and residence as enabling factors. The household wealth index indicates the economic ability to access services, while exposure to mass media functions as a crucial enabling factor by disseminating maternal health information, shaping health-seeking behaviors, and addressing knowledge deficiencies, especially in resource-constrained environments. Access to health care and residence encompasses geographic and structural impediments to service utilization. Need factors encompass both perceived and assessed health status, indicating the actual or subjective requirement for medical attention [21]. Termination of pregnancy and desired pregnancy were chosen as need factors because termination of pregnancy indicates obstetric history that heightens the risks of future pregnancy complications, whereas pregnancy intention affects the motivation to pursue and complete the continuum of maternal care. In this study, the classification and definitions of analytical variables adhered closely to the established schema, ensuring conceptual coherence. Figure 1 shows how these areas interact with each other, as described in the Andersen Behavioral Model. It shows how combinations of predisposing, enabling, and need factors affect the use of CoC services and, in the end, maternal and child health outcomes.

### Outcome Variable

The outcome variable is maternal dropout from CoC, which consists of dropping out from three integral components, which are (1) at least 4 ANC visits by skilled care providers during pregnancy, (2) delivery assisted by SBAs, and (3) at least 1 PNC visit by skilled care providers within 48 hours of delivery [14]. The skilled care providers in this aspect are designated as doctors, nurses, midwives, paramedics, community SBAs, and subassistant community medical officers. A mother was considered to have dropped out from the full CoC if she had not received any of these components. The mothers were assigned code 1 if they received all 3 components of the CoC; otherwise, code 0. Similarly, mothers who utilized any specific component of the CoC were assigned a code 1, otherwise 0, and analyzed separately.

### Covariates

We considered covariates, including age at delivery (<19 y, 19-30 y, and 31‐49 y); education level (no education, primary, secondary, and higher); husband’s education level (no education, primary, secondary, and higher); occupation (not working and working); husband’s occupation (not working and working); parity (1, 2‐3, and >3); ever-terminated pregnancy (never/no and ever/yes); desired pregnancy (yes and no); wealth index (poorest, poorer, middle, richer, and richest); any exposure to television, radio, or news (yes and no); facing problems such as getting permission and money and long distance to access health care (big problem and not a big problem); religion (Muslim and other); place of residence (rural and urban); division of residence (Barisal, Chittagong, Dhaka, Khulna, Mymensingh, Rajshahi, Rangpur, and Sylhet); and survey round (BDHS 2017‐2018 and BDHS 2022). These covariates were categorized based on how they align with the Andersen Behavioral Model (Figure 1).

### Statistical Analysis

The distribution of covariates by maternal dropouts from CoC was examined using cross-tabulations in a frequency distribution table, and the differences among 4 individual components were observed using the *χ*^2^ test.

The association between prevalence of dropout from maternal CoC and covariates was analyzed using logistic regression models. Four models were utilized based on the outcome variables, including dropout from (1) 4 ANC, (2) delivery assisted by SBAs, (3) PNC within 48 hours of delivery, and (4) all CoC components. Given that the selection of variables was guided by a theoretical framework, the Andersen Behavioral Model of Health Services, no covariate was excluded in the multivariable models, irrespective of the level of significance of the association in the univariable models.

Data from BDHS 2017‐2018 and BDHS 2022 were pooled, and a binary indicator for survey round was included in all models. We fitted survey-weighted logistic regression models for each stage of the CoC (completion of adequate ANC, institutional delivery, and PNC contact) as well as overall CoC completion, with socioeconomic and demographic characteristics as predictors. To assess whether the patterns of dropout and the associations between maternal characteristics and CoC engagement differed between survey rounds, we included interaction terms between survey round and each key predictor (eg, wealth quintile, education level, and place of residence). Equality of the survey-specific effects across socioeconomic and demographic factors was evaluated using joint Wald tests of the interaction coefficients; statistical significance indicated that the log odds ratios (ie, the strength and direction of associations) differed between BDHS 2017‐2018 and BDHS 2022 [22].

To ensure the accuracy of the estimates, the analyses were adjusted for sampling weight, strata, and primary sampling units. SEs of the odds ratio and variance inflation factors were observed to examine the multicollinearity. An SE of the odds ratio >2 was considered to indicate multicollinearity among the covariates [23]. All the statistical tests were conducted considering *P*<.05 as the significance level. The software STATA 17 (StataCorp, College Station) was used for data analysis. The study is reported in accordance with the STROBE (Strengthening the Reporting of Observational Studies in Epidemiology) guidelines for observational studies (Checklist 1).

### Ethical Considerations

The Demographic and Health Survey received approval from the ICF Macro Institutional Review Board, United States, which complies with all the criteria of 45 CFR 46 “Protection of Human Subjects.” As the data were collected in Bangladesh, the BDHS received ethical approval from the National Research Ethics Committee of the Bangladesh Medical Research Council, Dhaka, Bangladesh. Before being enrolled in the study, verbal informed agreement was obtained from each participant and their intimate partners (all ever-married women aged 15‐49 y). If the respondents were incapable of reading, verbal consent was considered the most appropriate method to confirm participation.

All survey data were anonymized before being made available for analysis, and no financial or other incentives were offered to participants for their participation or for the use of their data in this study. The manuscript and its supplementary materials do not include any information or images that could lead to the identification of individual respondents. Comprehensive descriptions of the ethical procedures and informed consent process are provided in the BDHS documentation and official reports.

## Results

**Figure 2.**
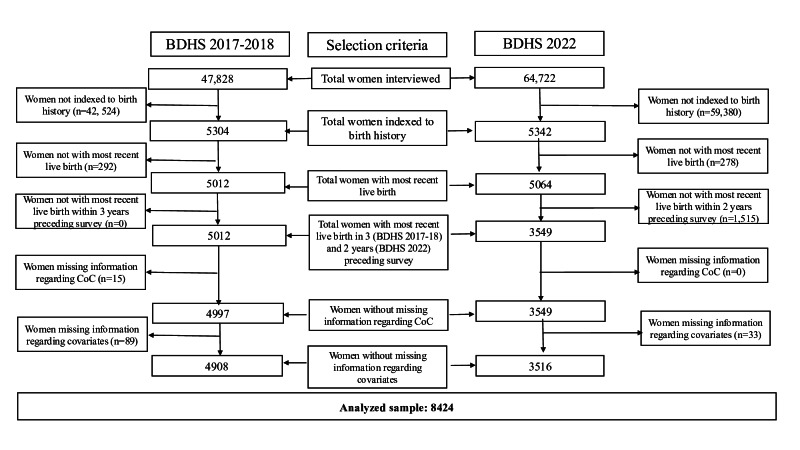
The sample selection process. BDHS: Bangladesh Demographic and Health Surveys; CoC: continuum of care.

**Figure 3.**
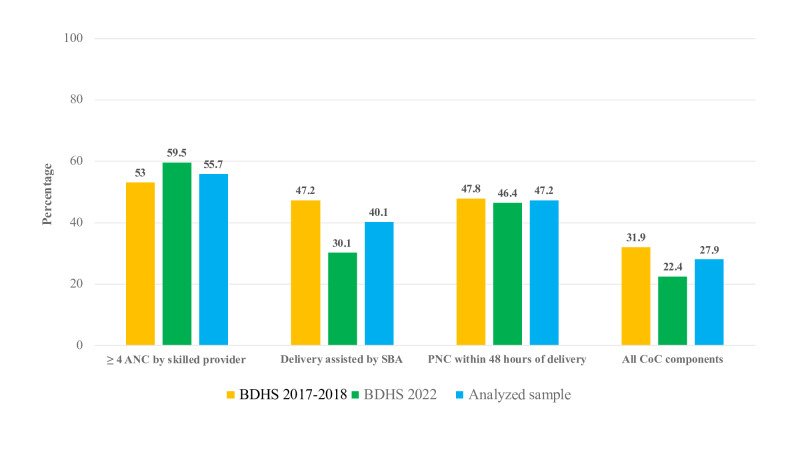
Maternal dropout from the continuum of care (CoC) and its component. ANC: antenatal care; BDHS: Bangladesh Demographic and Health Surveys; PNC: postnatal care; SBA: skilled birth attendant.

The sample selection process for this study is illustrated in Figure 2. In this study, 8424 mothers with complete information about outcome and covariates were analyzed. In the analyzed sample, maternal dropouts from having ≥4 ANC, delivery assisted by SBAs, PNC within 48 hours of delivery, and full CoC were 55.7% (n=4962), 40.1% (n=3378), 47.2% (n=3976), and 27.9% (n=2350), respectively (Figure 3).

Table 1 demonstrates the difference in dropouts from individual and all CoC components across BDHS 2017‐2018 and 2022 and shows clear changes over time. Across both BDHS rounds, dropouts from the maternal CoC remained high, with particularly large attrition among socioeconomically disadvantaged and rural women. The dropout at each stage (≥4 ANC visits, SBA-assisted delivery, and ≥4 PNC contacts) was consistently higher among women with little or no education, from poorer households, without media exposure, reporting major problems in accessing care, and residing in rural or underserved divisions. Educational and wealth gradients were steep: women with no schooling or in the poorest quintile had roughly doubled the dropout from full CoC compared with highly educated or richest women in both surveys. Although some absolute levels improved between 2017‐2018 and 2022, the pattern of persistent socioeconomic and geographic inequities in CoC coverage remained the dominant feature of the data.

**Table 1.** Association between the coverage of ≥4 antenatal care (ANC), postnatal care (PNC), and delivery by skilled birth attendants (SBAs), and full continuum of care (CoC) by maternal characteristics in Bangladesh according to the Bangladesh Demographic and Health Surveys (BDHS) survey 2017‐2018 (N=4908) and 2022 (N=3516).

Covariates	Dropout from ANC		Dropout from SBA-assisted delivery		Dropout from PNC		Dropout from all CoC components	
	BDHS 2017‐2018, n (%)	BDHS 2022, n (%)	BDHS 2017‐2018, n (%)	BDHS 2022, n (%)	BDHS 2017‐2018, n (%)	BDHS 2022, n (%)	BDHS 2017‐2018, n (%)	BDHS 2022, n (%)
Predisposing factors								
Age at delivery, y	*P*=.03	*P*=.002	*P*=.49	*P*=.23	*P*=.72	*P*=.18	*P*=.03	*P*=.03
<19	425 (52)	290 (63)	385 (47)	142 (31)	385 (47)	214 (46)	248 (30)	1060 (31)
19-30	1745 (59)	1442 (59)	1586 (46)	711 (29)	1831 (47)	1120 (46)	1060 (31)	1060 (31)
31-49	371 (56)	317 (53)	319 (49)	195 (32)	339 (52)	300 (50)	1060 (31)	1060 (31)
Education level	*P*<.001	*P*<.001	*P*<.001	*P*<.001	*P*<.001	*P*<.001	*P*<.001	*P*<.001
No education	241 (79)	125 (77)	223 (73)	88 (54)	222 (73)	109 (67)	184 (61)	70 (43)
Primary	898 (66)	590 (73)	897 (66)	378 (47)	900 (66)	489 (61)	648 (48)	292 (36)
Secondary	1138 (48)	1097 (59)	1019 (43)	523 (28)	1023 (44)	823 (44)	634 (27)	371 (20)
Higher	264 (30)	237 (34)	151 (17)	59 (9)	164 (18)	213 (31)	78 (9)	42 (6)
Husbands’ education	*P*<.001	*P*<.001	*P*<.001	*P*<.001	*P*<.001	*P*<.001	*P*<.001	*P*<.001
No education	475 (70)	373 (71)	469 (69)	261 (50)	468 (69)	343 (65)	358 (53)	205 (39)
Primary	1017 (62)	714 (60)	970 (59)	413 (40)	973 (59)	554 (54)	660 (40)	309 (30)
Secondary	783 (48)	686 (57)	682 (42)	291 (24)	941 (58)	497 (41)	437 (27)	206 (17)
Higher	266 (28)	276 (36)	169 (18)	83 (11)	773 (19)	240 (31)	89 (9)	55 (7)
Parity	*P*<.001	*P*<.001	*P*<.001	*P*<.001	*P*<.001	*P*<.001	*P*<.001	*P*<.001
1	821 (44)	733 (54)	643 (35)	276 (20)	656 (35)	806 (60)	399 (22)	194 (14)
2‐3	1286 (52)	1082 (59)	1229 (50)	592 (32)	1236 (50)	874 (47)	808 (33)	445 (24)
>3	434 (73)	234 (72)	418 (70)	180 (56)	417 (70)	215 (67)	337 (56)	136 (42)
Religion	*P*=.003	*P*<.001	*P*<.001	*P*<.001	*P*<.001	*P*<.001	*P*<.001	*P*<.001
Muslim	2355 (52)	1917 (59)	2136 (48)	1004 (31)	2342 (50)	1545 (48)	1450 (32)	744 (23)
Others	186 (45)	132 (47)	154 (37)	44 (16)	257 (62)	89 (32)	94 (23)	31 (11)
Division	*P*<.001	*P*<.001	*P*<.001	*P*<.001	*P*<.001	*P*<.001	*P*<.001	*P*<.001
Barisal	314 (60)	240 (64)	262 (50)	124 (33)	265 (51)	181 (49)	203 (39)	101 (27)
Chittagong	485 (59)	339 (58)	423 (52)	180 (31)	400 (49)	266 (45)	295 (36)	138 (23)
Dhaka	336 (46)	285 (53)	271 (37)	130 (24)	283 (39)	247 (46)	180 (25)	100 (19)
Khulna	202 (40)	224 (56)	175 (35)	50 (13)	173 (34)	115 (29)	90 (18)	37 (9)
Mymensingh	308 (52)	229 (51)	326 (55)	186 (41)	326 (55)	253 (56)	204 (34)	118 (26)
Rajshahi	257 (50)	211 (60)	221 (43)	88 (25)	221 (43)	144 (41)	140 (27)	61 (17)
Rangpur	209 (38)	258 (62)	262 (48)	135 (33)	264 (48)	206 (50)	138 (25)	87 (21)
Sylhet	430 (63)	263 (66)	378 (56)	155 (39)	377 (55)	222 (55)	294 (43)	133 (33)
Enabling factors								
Occupation	*P*=.31	*P*=.21	*P*<.001	*P*<.001	*P*<.001	*P*=.07	*P*<.001	*P*=.002
Not working	1518 (51)	1554 (58)	1217 (41)	731 (27)	1233 (42)	1229 (46)	842 (28)	561 (21)
Working	1023 (53)	495 (60)	1073 (55)	317 (39)	1076 (56)	405 (49)	702 (36)	214 (26)
Husbands’ occupation	*P*=.15	*P*=.10	*P*=.02	*P*=.02	*P*=.05	*P*=.22	*P*=.49	*P*=.19
Not working	24 (63)	40 (49)	11 (29)	15 (19)	12 (32)	43 (53)	10 (26)	13 (16)
Working	2517 (52)	2009 (58)	2279 (47)	1033 (30)	2297 (47)	1591 (46)	1534 (32)	762 (22)
Wealth index	*P*<.001	*P*<.001	*P*<.001	*P*<.001	*P*<.001	*P*<.001	*P*<.001	*P*<.001
Poorest	736 (69)	540 (75)	772 (73)	388 (54)	769 (72)	471 (66)	572 (54)	297 (41)
Poorer	610 (62)	504 (69)	594 (60)	279 (38)	598 (60)	386 (53)	406 (41)	212 (29)
Middle	459 (52)	442 (62)	471 (54)	199 (28)	409 (46)	320 (45)	253 (29)	143 (20)
Richer	452 (46)	345 (50)	356 (37)	126 (18)	368 (38)	252 (37)	225 (23)	91 (13)
Richest	284 (28)	218 (33)	159 (16)	56 (8)	165 (17)	205 (31)	88 (9)	32 (5)
Media exposure^a^	*P*<.001	*P*<.001	*P*<.001	*P*<.001	*P*<.001	*P*<.001	*P*<.001	*P*<.001
No exposure	1186 (67)	1080 (70)	1138 (65)	626 (40)	1129 (64)	847 (55)	852 (48)	498 (32)
Any exposure	1355 (43)	969 (49)	1152 (37)	422 (21)	1180 (37)	787 (40)	692 (22)	277 (14)
Accessing health care^b^	*P*<.001	*P*<.001	*P*<.001	*P*<.001	*P*<.001	*P*<.001	*P*<.001	*P*<.001
Not a problem	875 (42)	740 (52)	766 (37)	358 (25)	781 (37)	608 (43)	469 (23)	253 (18)
Big problem	1666 (59)	1309 (63)	1524 (54)	690 (33)	1528 (54)	1026 (49)	1075 (38)	522 (25)
Residence	*P*<.001	*P*<.001	*P*<.001	*P*<.001	*P*<.001	*P*<.001	*P*<.001	*P*<.001
Urban	687 (41)	503 (44)	561 (33)	205 (18)	575 (34)	432 (38)	359 (21)	147 (13)
Rural	1854 (58)	1546 (65)	1729 (54)	843 (36)	1734 (54)	1202 (51)	1185 (37)	628 (27)
Need factors								
Terminated pregnancy	*P*=.02	*P*=.002	*P*=.02	*P*=.07	*P*=.009	*P*=.33	*P*=.09	*P*=.09
No/never	2138 (53)	1712 (59)	2142 (53)	877 (30)	2122 (52)	1349 (47)	1302 (32)	655 (23)
Yes/ever	403 (48)	337 (53)	476 (57)	171 (27)	477 (57)	285 (45)	242 (29)	120 (19)
Desired pregnancy	*P*<.001	*P*=.02	*P*<.001	*P*<.001	*P*<.001	*P*<.001	*P*<.001	*P*<.001
Yes	1975 (50)	1618 (57)	1718 (44)	781 (28)	1734 (45)	1556 (55)	1130 (29)	580 (21)
No	616 (59)	431 (62)	572 (55)	267 (38)	575 (55)	326 (47)	414 (40)	195 (28)

a Media exposure: newspaper or television or radio.

b Problem to access health care: permission to go, monetary constraints, or distance to health facilities.

Table 2 shows the adjusted odds ratios (aORs) for factors that are linked to dropping out of all parts of the maternal CoC. It compares BDHS 2017‐2018 and BDHS 2022. Joint Wald tests of the survey-round×covariate interactions indicated significant disparities in the associations for various key predictors between the 2 survey rounds.

**Table 2.** Factors associated with dropouts from the continuum of care among the mothers in Bangladesh from 2017 to 2022.

Covariates	BDHS^a^ 2017‐18		BDHS 2022		Pooled		
	AOR^b^ (95% CI)	*P* value	AOR (95% CI)	*P* value	AOR (95% CI)	*P* value	*P* value^c^
Predisposing factors							
Age at delivery (years) (Reference: <19 y)	1	—^d^	1	—	1	—	<.001
19-30	0.72 (0.57-0.90)	.004	0.82 (0.57-1.18)	.29	0.75 (0.62-0.91)	.004	
31-49	0.49 (0.34-0.69)	<.001	0.68 (0.41-1.14)	.15	0.56 (0.42-0.74)	<.001	
Education level (Reference: higher)	1	—	1	—	1	—	<.001
Secondary	1.49 (1.10-2.01)	.009	1.65 (1.10-2.47)	.02	1.52 (1.19-1.94)	.001	
Primary	2.17 (1.52-3.10)	<.001	2.28 (1.46-3.55)	<.001	2.17 (1.64-2.87)	<.001	
No education	3.20 (2.12-4.82)	<.001	2.16 (1.22-3.81)	.008	2.70 (1.94-3.77)	<.001	
Husband’s education (Reference: higher)	1	—	1	—	1	—	<.001
Secondary	1.63 (1.19-2.23)	.003	1.47 (1.01-2.15)	.04	1.59 (1.25-2.03)	<.001	
Primary	1.84 (1.34-2.53)	<.001	1.98 (1.32-2.96)	.001	1.91 (1.48-2.44)	<.001	
No education	1.20 (1.38-2.87)	<.001	2.21 (1.46-3.34)	<.001	2.09 (1.59-2.76)	<.001	
Parity (Reference: 1)	1	—	1	—	1	—	<.001
2‐3	1.60 (1.30-1.95)	<.001	1.73 (1.36-2.20)	<.001	1.64 (1.40-1.91)	<.001	
>3	2.79 (2.04-3.82)	<.001	2.61 (1.70-4.00)	<.001	2.73 (2.12-3.50)	<.001	
Religion (Reference: others)	1	—	1	—	1	—	<.001
Muslim	1.50 (1.08-2.07)	.02	2.42 (1.39-4.23)	.002	1.78 (1.33-2.38)	<.001	
Division (Reference: Dhaka)	1	—	1	—	1	—	<.001
Barisal	1.03 (0.71-1.49)	.88	1.20 (0.78-1.86)	.40	1.11 (0.83-1.48)	.49	
Chittagong	1.34 (0.96-1.85)	.08	1.09 (0.75-1.59)	.66	1.23 (0.96-1.57)	.10	
Khulna	0.47 (0.32-0.71)	<.001	0.40 (0.25-0.66)	.000	0.46 (0.34-0.62)	<.001	
Mymensingh	0.85 (0.57-1.24)	.39	0.83 (0.55-1.26)	.39	0.84 (0.63-0.11)	.23	
Rajshahi	0.78 (0.56-1.09)	.15	0.82 (0.54-1.25)	.36	0.79 (0.61-1.02)	.08	
Rangpur	0.51 (0.34-0.77)	.002	0.71 (0.45-1.10)	.13	0.59 (0.44-0.80)	.001	
Sylhet	1.26 (0.85-1.86)	.25	1.34 (0.92-1.96)	.13	1.31 (0.98-1.74)	.07	
Enabling factors							
Occupation (Reference: working)	1	—	1	—	1	—	.41
Not working	0.94 (0.80-1.10)	.42	0.91 (0.72-1.16)	.46	0.93 (0.81-1.06)	.30	
Husbands’ occupation (Reference: working)	1	—	1	—	1	—	.95
Not working	1.30 (0.59-2.85)	.51	1.04 (0.47-2.32)	.91	1.07 (0.60-1.92)	.81	
Wealth index (Reference: richest)	1	—	1	—	1	—	<.001
Poorest	4.08 (2.82-5.89)	<.001	3.93 (2.40-6.42)	<.001	4.04 (3.02-5.41)	<.001	
Poorer	3.38 (2.39-4.78)	<.001	3.10 (2.01-5.09)	.001	3.32 (2.53-4.35)	<.001	
Middle	2.41 (1.71-3.38)	<.001	2.22 (1.40-3.54)	.001	2.34 (1.79-3.06)	<.001	
Richer	2.16 (1.56-2.99)	<.001	1.83 (1.13-2.96)	.01	2.05 (1.57-2.67)	<.001	
Media exposure^e^ (Reference: any exposure)	1	—	1	—	1	—	<.001
No exposure	1.61 (1.33-1.95)	<.001	1.60 (1.32-1.95)	<.001	1.58 (1.38-1.82)	<.001	
Accessing health care^f^ (Reference: big problem)	1	—	1	—	1	—	.02
Not a big problem	0.84 (0.71-1.00)	.05	0.92 (0.75-1.11)	.36	0.87 (0.76-0.99)	.04	
Residence (Reference: urban)	1	—	1	—	1	—	<.001
Rural	1.40 (1.12-1.72)	.003	1.44 (1.08-1.94)	.01	1.40 (1.18-1.67)	<.001	
Need factors							
Terminated pregnancy (Reference: no/never)	1	—	1	—	1	—	<.001
Yes/ever	0.79 (0.65-0.95)	.02	0.67 (0.51-0.87)	.003	0.74 (0.63-0.86)	<.001	
Desired pregnancy (Reference: yes/then)	1	—	1	—	1	—	.07
No	1.36 (1.12-1.66)	.002	1.12 (0.87-1.42)	.34	1.27 (1.09-1.48)	.002	
Survey round (Reference: BDHS 2017‐2018)	—	—	—	—	1	—	—
BDHS 2022	—	—	—	—	0.59 (0.51-0.69)	<.001	

a BDHS: Bangladesh Demographic and Health Surveys.

b AOR: adjusted odds ratio.

c *P* value obtained from joint Wald tests of the interaction coefficients between survey years and covariates.

d Not applicable.

e Media exposure: newspaper, television, or radio.

f Problem to access health care: permission to go, monetary constraints, or distance to health facilities.

The impacts of age at delivery, maternal education, husband’s education, parity, religion, division, wealth index, media exposure, health care access, residence, terminated pregnancy, and desired pregnancy exhibited significant variations between survey rounds. Conversely, the associations with maternal occupation (*P*=.40) and husband’s occupation (*P*=.95) exhibited no significant differences between BDHS 2017‐2018 and BDHS 2022, indicating consistent patterns for these variables over time.

Some important patterns that were specific to the survey are as follows: the protective effect of older age at delivery was stronger in 2017‐2018 (AOR 0.49, 95% CI 0.34-0.69 for 31–49 years) than in 2022 (AOR 0.68, 95% CI 0.41-1.14); the aOR for Muslim religion went up from 1.50 (95% CI 1.08-2.07) in 2017‐2018 to 2.42 (95% CI 1.39-4.23) in 2022; and women with no education were at a greater disadvantage in 2017‐2018 (AOR 3.20, 95% CI 2.12-4.82) than in 2022 (AOR 2.16, 95% CI 1.22-3.81), which suggests that the education gradient is getting smaller. The survey round main effect shows that the odds of dropping out are much lower in BDHS 2022 than in 2017‐2018 (AOR 0.59, 95% CI 0.51‐0.69, *P*<.001). This means that the completion of the CoC has improved over time.

In the supplementary materials, we presented the factors associated with dropouts from ANC, SBA, and PNC, separately (see Tables 1, 2, and 3 in Multimedia Appendix 1). In all the regression models, SEs of the odds ratio for all covariates were <2, indicating an acceptable level of multicollinearity among the independent variables. Furthermore, the variance inflation factor of the covariates was less than 3 for both the adjusted and unadjusted models (see Table 4 in Multimedia Appendix 2).

## Discussion

### Principal Findings

This study investigated the trends in the CoC over the past 5 years and analyzed the factors contributing to dropouts from CoC in Bangladesh, which include ANC, skilled birth attendance, and PNC. Using the Andersen Healthcare Utilization Model, we discovered that predisposing factors (age, education level of the respondent and her husband, parity, desired pregnancy, religion, and survey round), enabling factors (occupation of the respondent and her husband, wealth index, and media exposure), and need factors (desired pregnancy and terminated pregnancy) increased the likelihood of maternal dropout from CoC. From BDHS 2017‐2018 (31.9%) to BDHS 2022 (22.4%), there was a significant drop in the overall dropout from CoC. This suggests that maternal health service utilization has gotten a little better.

The statistically significant differences noted in the associations between covariates and maternal CoC dropouts across the 2 survey rounds (*P*<.05 in the interaction test) illustrate Bangladesh’s evolving maternal health landscape from 2017 to 2022, characterized by policy reforms and unprecedented disruptions in the health system. During this time between surveys, Bangladesh put the National Strategy for Maternal Health 2019‐2030 into action. This plan focused on making it easier for everyone to get maternal health services and improving community-based care delivery [24]. The COVID-19 pandemic (2020‐2022) significantly disrupted maternal health service provision and utilization, with studies indicating approximately 30% reductions in ANC, institutional deliveries, and postnatal care in 2020, followed by an incomplete recovery by 2021 [25]. This disruption had a bigger impact on vulnerable groups, such as women from lower wealth quintiles, people who live in rural areas, and people with little education. This could make existing inequalities worse. The substantial interaction effects for enabling factors, including wealth index (*P*<.001), media exposure (*P*<.001), health care access barriers (*P*=.02), and residence (*P*<.001), indicate that the protective or risk-enhancing roles of these determinants fluctuated between survey rounds. This likely reflects the health system’s varying capacity to serve different population subgroups during the pandemic and the inconsistent pace of postpandemic recovery across socioeconomic strata [26-28].

These changes in CoC dropout patterns over time can be explained by a number of interconnected barriers. Lower maternal education is frequently correlated with diminished financial independence and decision-making abilities, which subsequently adversely impacts the continuity of health care services for both the mother and her children [29]. The COVID-19 pandemic made financial problems worse. Studies showed that household income dropped by 19% to 20%, and access to maternal health care was disrupted [25,30]. As the cost of living went up, there were more demands on limited household resources. For example, maintaining continuity through the CoC requires multiple visits to health care facilities. This requirement competes with time for household duties and leads to high indirect medical costs, such as transportation and medications [26]. Women from lower socioeconomic backgrounds in Bangladesh bear a disproportionate share of health care financing [31]. This makes the burden of out-of-pocket costs even heavier for them.

To address dropout from maternal health services, Bangladesh has implemented social health protection schemes, such as Shasthyo Surokhsha Karmasuchi and Maternal Health Voucher Scheme, targeting disadvantaged populations [32]. However, evidence suggests that these programs typically fail to monitor the complete maternal CoC, resulting in women discontinuing service uptake after initial contact with 1 component of the continuum [33]. This programmatic limitation highlights the critical gap between initiating maternal health care and maintaining continuity across all phases of pregnancy, delivery, and postpartum care.

Moreover, the notable temporal fluctuations in need factors, especially desired pregnancy status (*P*=.07) and history of terminated pregnancies (*P*<.001), may indicate evolving reproductive health behaviors and family planning dynamics amid this era of economic instability and service interruption [34]. The temporal fluctuations in the strength of associations concerning predisposing factors, such as age at delivery, parity, religion, and division, signify the changing socioeconomic and geographic determinants of maternal health care utilization. The significant interaction for division (*P*<.001) highlights enduring geographic disparities in maternal health care access, with regions such as Khulna and Rangpur exhibiting improved outcomes, whereas others encountered heightened obstacles during the intersurvey period. These ongoing geographic disparities, along with the ongoing difficulty of keeping CoC continuity across all parts, show that Bangladesh needs customized, area-specific interventions to make the full maternal health care pathway stronger [35].

Moreover, the high rate of unintended pregnancies, which are frequently identified late, along with related effects, such as anxiety and depression, discourages women from obtaining CoC in Bangladesh. In addition, late detection inhibits mothers from commencing ANC punctually, resulting in inadequate access to the prescribed number of ANC visits. Our finding that women with unwanted pregnancies had higher odds of dropping out of the CoC is consistent with previous literature linking unintended pregnancy to lower utilization of ANC, skilled delivery, and postnatal services in Bangladesh and other low- and middle-income countries [36,37]. This pattern does not merely indicate a lack of motivation; it likely signifies the presence of multiple intersecting disadvantages. Women with unintended pregnancies are more prone to encounter financial constraints, restricted decision-making authority, stigma or fear of judgment from health care providers, and symptoms of depression or stress, all of which can hinder timely and sustained engagement with services [36].

The study utilized the latest 2 consecutive nationally representative surveys, with high response rates. Therefore, these datasets allow for reliable conclusions about maternal health care utilization patterns across the country within the defined time. In addition, the selection of factors is guided by the Andersen Behavioral Model of Health Care Services, providing a more precise estimate. The evaluation of outcomes and factors followed validated measurements, minimizing the risk of assessment bias. In contrast to the strengths of the study, there remain some challenges to interpreting the findings. First, the cross-sectional design of the study limits establishing a causal nature of the association found between identified factors and CoC components. Second, the possibility of recall bias may affect the accuracy of findings, as the recall period spanned up to 3 years in the datasets. Third, the analyses were restricted to women with complete information on CoC utilization and all covariates, which introduces the possibility of selection bias if women with missing data differ systematically from those included in the analytic sample. Finally, the study did not explore health care system–related factors, such as health care providers’ attitudes, patient-provider relationships, availability of workforce, quality of care, and factors underlying the decision-making dynamics of women to uptake CoC services. These contextual elements are critical to fully understanding health care utilization.

This study offers crucial insights into the utilization of CoC in maternal health care, emphasizing the importance of policy support to improve the maternal utilization of CoC, the development of relevant interventions, and the provision of services.

### Conclusion

In this study of 8424 mothers, more than 1 in 4 failed to complete the continuum of maternal care, with dropout most pronounced at the ANC stage. Dropout was strongly associated with lower education, higher parity, lower wealth, rural residence, and younger maternal age and was also influenced by media exposure, pregnancy intention, and religious affiliation, underscoring the complex interplay of sociodemographic and experiential factors in maternal health service utilization.

## Supplementary material

10.2196/85875 Multimedia Appendix 1 Factors associated with dropout from antenatal care, skilled birth attendant delivery, and postnatal care.

10.2196/85875 Multimedia Appendix 2 Fitness of regression models.

10.2196/85875 Checklist 1 STROBE checklist.

## Abbreviations

ANC: antenatal care
AOR: adjusted odds ratio
BDHS: Bangladesh Demographic and Health Surveys
CoC: continuum of care
PNC: postnatal care
SBA: skilled birth attendant
STROBE: Strengthening the Reporting of Observational Studies in Epidemiology
