# Supplementary material for: Uncovering the Reasons Behind Maternal Care Dropout in Bangladesh: Cross-Sectional Study
Source: JMIR Public Health Surveill. 2026 Apr 1;12:e85875. doi: 10.2196/85875 (PMC13043009; doi:10.2196/85875)
Supplement: Multimedia Appendix 1 [file publichealth-v12-e85875-s001.docx]

Supplementary Table 1: Factors associated with dropout from ANC

| Covariates | BDHS 2017-18 | | BDHS 2022 | | Pooled | | |
| --- | --- | --- | --- | --- | --- | --- | --- |
|  | AOR (95% CI) | p value | AOR (95% CI) | p value | AOR (95% CI) | p value | p value^a^ |
| ***Predisposing factors*** | | | | | | | |
| **Age at delivery** |  |  |  |  |  |  |  |
| < 19y | ref |  | ref |  | ref |  | 0.011 |
| 19-30y | 0.88 (0.70, 1.10) | 0.255 | 0.98 (0.75, 1.27) | 0.870 | 0.92 (0.78, 1.09) | 0.362 |  |
| 31-49y | 0.85 (0.61, 1.19) | 0.346 | 0.72 (0.50, 1.02) | 0.067 | 0.78 (0.61, 0.99) | 0.045 |  |
| **Education level** |  |  |  |  |  |  |  |
| Higher | ref |  | ref |  | ref |  | <0.001 |
| Secondary | 1.11 (0.88, 1.40) | 0.372 | 1.48 (1.13, 1.93) | 0.004 | 1.25 (1.04, 1.49) | 0.014 |  |
| Primary | 1.45 (1.10, 1.94) | 0.011 | 2.20 (1.56, 3.10) | 0.000 | 1.72 (1.39, 2.15) | 0.000 |  |
| No education | 2.596 (1.75, 3.85) | 0.000 | 2.74 (1.57, 4.77) | 0.000 | 2.72 (1.95, 3.80) | 0.000 |  |
| **Husbands' education** |  |  |  |  |  |  |  |
| Higher | ref |  | ref |  | ref |  | <0.001 |
| Secondary | 1.61 (1.26, 2.04) | 0.000 | 1.42 (1.10, 1.83) | 0.007 | 1.51(1.28,1.81) | 0.000 |  |
| Primary | 2.06 (1.56, 2.72) | 0.000 | 1.63 (1.21, 2.11) | 0.001 | 1.84 (1.50, 2.24) | 0.000 |  |
| No education | 2.04 (1.48, 2.80) | 0.000 | 1.30 (.94, 1.81) | 0.113 | 1.64 (1.30, 2.07) | 0.000 |  |
| **Parity** |  |  |  |  |  |  |  |
| 1 | ref |  | ref |  | ref |  | <0.001 |
| 2-3 | 1.18 (0.99, 1.40) | 0.072 | 1.17 (0.96, 1.42) | 0.104 | 1.17 (1.03, 1.33) | 0.019 |  |
| >3 | 1.67 (1.22, 2.29) | 0.001 | 1.68 (1.12, 2.53) | 0.012 | 1.73 (1.35, 2.21) | 0.000 |  |
| **Religion** |  |  |  |  |  |  |  |
| Others | ref |  | ref |  | ref |  | 0.007 |
| Muslim | 1.24 (0.93,1.64) | 0.138 | 1.56 (1.05, 2.33) | 0.028 | 1.40 (1.09, 1.80) | 0.008 |  |
| **Division** |  |  |  |  |  |  |  |
| Dhaka | ref |  | ref |  | ref |  | <0.001 |
| Barisal | 1.04 (0.74, 1.46) | 0.820 | 1.40 (0.95, 2.07) | 0.090 | 1.20 (0.92,1.55) | 0.173 |  |
| Chattogram | 1.41 (1.06, 1.86) | 0.016 | 0.94 (0.69, 1.29) | 0.728 | 1.20 (0.97, 1.48) | 0.096 |  |
| Khulna | 0.61 (0.44, 0.85) | 0.004 | 1.19 (0.86, 1.65) | 0.302 | 0.84 (0.66, 1.06) | 0.146 |  |
| Mymensingh | 0.76 (0.53, 1.10) | 0.147 | 0.55 (0.37, 0.82) | 0.003 | 0.68 (0.52, 0.89) | 0.005 |  |
| Rajshahi | 0.86 (0.62, 1.11) | 0.377 | 1.33 (0.88, 2.01) | 0.169 | 1.04 (0.81, 1.34) | 0.750 |  |
| Rangpur | 0.40 (0.28, 0.57) | 0.000 | 1.11 (0.76, 1.63) | 0.571 | 0.64 (0.50, 0.83) | 0.001 |  |
| Sylhet | 1.16 (0.81, 1.65) | 0.415 | 1.11 (0.78, 1.57) | 0.559 | 1.17 (1.90, 1.51) | 0.234 |  |
| ***Enabling factors*** | | | | | | | |
| **Occupation** |  |  |  |  |  |  |  |
| Working | ref |  | ref |  | ref |  | 0.022 |
| Not working | 1.16 (0.10, 1.37) | 0.064 | 1.08 (0.89, 1.31) | 0.405 | 1.16 (1.03, 1.31) | 0.016 |  |
| **Husbands' occupation** |  |  |  |  |  |  |  |
| Working | ref |  | ref |  | ref |  | 0.162 |
| Not working | 2.03 (0.10, 4.14) | 0.052 | 1.26 (0.76, 2.11) | 0.367 | 1.35 (0.89,2.06) | 0.160 |  |
| **Wealth index** |  |  |  |  |  |  |  |
| Richest | ref |  | ref |  | ref |  | <0.001 |
| Poorest | 2.76 (2.00,3.798) | 0.000 | 2.50 (1.78, 3.52) | 0.000 | 2.56(2.03, 3.22) | 0.000 |  |
| Poorer | 2.63 (1.97, 3.51) | 0.000 | 2.29 (1.68, 3.12) | 0.000 | 2.41(1.96, 2.97) | 0.000 |  |
| Middle | 2.09 (1.58, 2.78) | 0.000 | 2.01 (1.51, 2.68) | 0.000 | 2.01(1.65, 2.45) | 0.000 |  |
| Richer | 1.83 (1.42, 2.36) | 0.000 | 1.42 (1.09, 1.85) | 0.008 | 1.64(1.37, 1.97) | 0.000 |  |
| **Media exposure^b^** |  |  |  |  |  |  |  |
| Any exposure | ref |  | ref |  | ref |  | <0.001 |
| No exposure | 1.47 (1.23, 1.75) | 0.000 | 1.51 (1.27, 1.80) | 0.000 | 1.46 (1.29, 1.65) | 0.000 |  |
| **Accessing healthcare^c^** |  |  |  |  |  |  |  |
| Big problem | ref |  | ref |  | ref |  | <0.001 |
| Not a big problem | 0.77 (0.66, 0.90) | 0.001 | 0.80 (0.67, 0.94) | 0.009 | 0.81 (0.72, 0.91) | 0.000 |  |
| **Residence** |  |  |  |  |  |  |  |
| Urban | ref |  | ref |  | ref |  | <0.001 |
| Rural | 1.31(1.07, 1.60) | 0.009 | 1.63 (1.31, 2.03) | 0.000 | 1.46 (1.26, 1.70) | 0.000 |  |
| ***Needs factors*** |  |  |  |  |  |  |  |
| **Terminated pregnancy** |  |  |  |  |  |  |  |
| No/never | ref |  | ref |  | ref |  | <0.001 |
| Yes/ever | 0.77 (0.64, 0.92) | 0.005 | 0.67 (.55,.83) | 0.000 | 0.73 (0.63, 0.83) | 0.000 |  |
| **Desired pregnancy** |  |  |  |  |  |  |  |
| Yes/then | ref |  | ref |  | ref |  | 0.008 |
| No | 1.35 (1.14, 1.59) | 0.001 | 1.08 (0.86, 1.35) | 0.519 | 1.22 (1.07,1.40) | 0.004 |  |
| ***Survey round*** | | | | | | | |
| BDHS 2017-18 |  |  |  |  | ref |  |  |
| BDHS 2022 |  |  |  |  | 1.36 (1.19, 1.56) | 0.000 | NA |

^a^P-value obtained from joint Wald tests of the interaction between survey years and covariates; ^b^Media exposure: newspaper or television or radio; ^c^Problem to access healthcare: permission to go or monetary constrains or distance to health facilities; NA (not applicable)

**Supplementary Table 2: Factors associated with dropout from SBA delivery**

|  | BDHS 2017-18 | | BDHS 2022 | | Pooled | | |
| --- | --- | --- | --- | --- | --- | --- | --- |
|  | AOR (95% CI) | p value | AOR (95% CI) | p value | AOR (95% CI) | p value | p value^a^ |
| ***Predisposing factors*** |  |  |  |  |  |  |  |
| **Age at delivery** |  |  |  |  |  |  |  |
| < 19y | ref |  | ref |  | ref |  | <0.001 |
| 19-30y | 0.73 (0.59, 0.91) | 0.005 | 0.76 (0.56, 1.04) | 0.087 | 0.74 (0.62, 0.89) | 0.001 |  |
| 31-49y | 0.45 (0.33, 0.61) | 0.000 | 0.64 (0.41, 1.00) | 0.051 | 0.53 (0.41, 0.69) | 0.000 |  |
| **Education level** |  |  |  |  |  |  |  |
| Higher | ref |  | ref |  | ref |  | <0.001 |
| Secondary | 1.48 (1.61, 1.87) | 0.001 | 1.79 (1.25, 2.57) | 0.001 | 1.58 (1.30, 1.93) | 0.000 |  |
| Primary | 2.34 (1.73, 3.18) | 0.000 | 2.48 (1.66, 3.71) | 0.000 | 2.37 (1.86, 3.02) | 0.000 |  |
| No education | 2.65 (1.77, 3.95) | 0.000 | 2.45 (1.40, 4.28) | 0.002 | 2.52 (1.82, 3.49) | 0.000 |  |
| **Husbands' education** |  |  |  |  |  |  |  |
| Higher | ref |  | ref |  | ref |  | <0.001 |
| Secondary | 1.63 (1.27, 2.10) | 0.000 | 1.32 (0.95, 1.83) | 0.094 | 1.53 (1.25, 1.87) | 0.000 |  |
| Primary | 1.96 (1.51, 2.56) | 0.000 | 1.79 (1.26, 2.55) | 0.001 | 1.91 (1.55, 2.36) | 0.000 |  |
| No education | 2.03 (1.46, 2.81) | 0.000 | 1.91 (1.32, 2.77) | 0.001 | 2.01 (1.58, 2.57) | 0.000 |  |
| **Parity** |  |  |  |  |  |  |  |
| 1 | ref |  | ref |  | ref |  | <0.001 |
| 2-3 | 1.83 (1.52, 2.21) | 0.000 | 1.72 (1.38, 2.13) | 0.000 | 1.77 (1.54, 2.04) | 0.000 |  |
| >3 | 2.78 (2.03, 3.80) | 0.000 | 2.89 (1.99, 4.21) | 0.000 | 2.80 (2.20, 3.56) | 0.000 |  |
| **Religion** |  |  |  |  |  |  |  |
| Others | ref |  | ref |  | ref |  | <0.001 |
| Muslim | 1.48 (1.09, 2.00) | 0.012 | 2.39 (1.50, 3.82) | 0.000 | 1.72 (1.33, 2.24) | 0.000 |  |
| **Division** |  |  |  |  |  |  |  |
| Dhaka | ref |  | ref |  | ref |  | <0.001 |
| Barisal | 0.80 (0.58, 1.11) | 0.191 | 1.19 (0​​.81, 1.75) | 0.375 | 0.96 (0.74, 1.24) | 0.737 |  |
| Chattogram | 1.15 (0.84, 1.57) | 0.389 | 1.12 (.79, 1.59) | 0.513 | 1.13 (0.89, 1.43) | 0.310 |  |
| Khulna | 0.63 (0.45, 0.87) | 0.006 | 0.37 (0.25, 0.57) | 0.000 | 0.53 (0.41, .69) | 0.000 |  |
| Mymensingh | 1.17 (0.84, 1.64) | 0.356 | 1.23 (0.87, 1.73) | 0.243 | 1.19 (0.94,1.52) | 0.154 |  |
| Rajshahi | 0.78 (0.56, 1.07) | 0.131 | 0..88 (0.60, 1.27) | 0.491 | 0.81 (0.63, 1.03) | 0.094 |  |
| Rangpur | 0.78 (0.54, 1.13) | 0.186 | 0.92 (0.62, 1.37) | 0.683 | 0.84 (0.64 ,1.10) | 0.199 |  |
| Sylhet | 1.25 (0.88, 1.77) | 0.202 | 1.27 (.89, 1.82) | 0.191 | 1.27 (0.98, 1.63) | 0.067 |  |
| ***Enabling factors*** | | | | | | | |
| **Occupation** |  |  |  |  |  |  |  |
| Working | ref |  | ref |  | ref |  | <0.001 |
| Not working | 0.78 (0.66, 0.91) | 0.002 | 0.69 (0.55, 0.87) | 0.002 | 0.75 (0.66, 0.85) | 0.000 |  |
| **Husbands' occupation** |  |  |  |  |  |  |  |
| Working | ref |  | ref |  | ref |  | 0.197 |
| Not working | 0.64 (0.31, 1.36) |  | 0.77 (0​​.36, 1.64) | 0.501 | 0.72 (0.41, 1.25) | 0.241 |  |
| **Wealth index** |  |  |  |  |  |  |  |
| Richest | ref |  | ref |  | ref |  | <0.001 |
| Poorest | 4.40 (3.19, 6.06) | 0.000 | 3.85 (2.52, 2.88) | 0.000 | 4.25 (3.31, 5.45) | 0.000 |  |
| Poorer | 3.35 (2.47,4.56) | 0.000 | 3.00 (2.00, 4.51) | 0.000 | 3.26 (2.57, 4.14) | 0.000 |  |
| Middle | 2.58 (1.95, 3.42) | 0.000 | 2.00 (1.35, 2.95) | 0.001 | 2.36 (1.89, 2.96) | 0.000 |  |
| Richer | 1.10 (1.54, 2.58) | 0.000 | 1.55 (1.00, 2.36) | 0.045 | 1.84 (1.47, 2.29) | 0.000 |  |
| **Media exposure^b^** |  |  |  |  |  |  |  |
| Any exposure | ref |  | ref |  | ref |  | <0.001 |
| No exposure | 1.43 (1.18, 1.74) | 0.000 | 1.35 (1.12, 1.62) | 0.001 | 1.38 (1.21, 1.58) | 0.000 |  |
| **Accessing healthcare^c^** |  |  |  |  |  |  |  |
| Big problem | ref |  | ref |  | ref |  | 0.270 |
| Not a big problem | 0.93 (0.80, 1.09) | 0.391 | 0..96 (.81, 1.14) | 0.664 | 0.94 (0.83, 1.05) | 0.283 |  |
| **Residence** |  |  |  |  |  |  |  |
| Urban | ref |  | ref |  | ref |  | <0.001 |
| Rural | 1.37 (1.11, 1.69) | 0.003 | 1.46 (1.133, 1.89) | 0.004 | 1.40 (1.19, 1.64) | 0.000 |  |
| ***Needs factors*** | | | | | | | |
| **Terminated pregnancy** |  |  |  |  |  |  |  |
| No/never | ref |  | ref |  | ref |  | <0.001 |
| Yes/ever | 0.74 (0.62, 0.88) | 0.001 | 0.66 (0.52, 0.84) | 0.001 | 0.71 (0.61, 0.81) | 0.000 |  |
| **Desired pregnancy** |  |  |  |  |  |  |  |
| Yes/then | ref |  | ref |  | ref |  | 0.018 |
| No | 1.23 (1.01,1.50) | 0.037 | 1.17 (0.93, 1.45) | 0.172 | 1.20 (1.04, 1.39) | 0.012 |  |
| ***Survey round*** | | | | | | | |
| BDHS 2017-18 |  |  |  |  | ref |  | NA |
| BDHS 2022 |  |  |  |  | 0.45 (0.39, 0.52) | 0.000 |  |

^a^P-value obtained from joint Wald tests of the interaction between survey years and covariates; ^b^Media exposure: newspaper or television or radio; ^c^Problem to access healthcare: permission to go or monetary constrains or distance to health facilities; NA (not applicable)

**Supplementary table 3: Factors associated with dropout from PNC**

| **Covariates** |  | | | | | | |
| --- | --- | --- | --- | --- | --- | --- | --- |
|  | **BDHS 2017-18** | | **BDHS 2022** | | **Pooled** | | |
|  | **AOR (95% CI)** | **p value** | **AOR (95% CI)** | **p value** | **AOR (95% CI)** | **p value** | **p value^a^** |
| ***Predisposing factors*** | | | | | | | |
| **Age at delivery** |  |  |  |  |  |  |  |
| < 19y | Ref |  | Ref |  | Ref |  | <0.001 |
| 19-30y | 0.75 (0.61, 0.93) | 0.010 | 0.96 (0.73, 1.25) | 0.756 | 0.84 (0.72, 0.99) | 0.042 |  |
| 31-49y | 0.47 (0.35, 0.63)) | 0.000 | 1.00 (0.70, 1.43) | 0.983 | 0.67 (0.54, 0.84) | 0.001 |  |
| **Education level** |  |  |  |  |  |  |  |
| Higher | Ref |  | Ref |  | Ref |  | <0.001 |
| Secondary | 1.37 (1.07, 1.74) | 0.011 | 1.28 (1.01, 1.62) | 0.043 | 1.32 (1.12, 1.57) | 0.001 |  |
| Primary | 2.19 (1.61, 2.98) | 0.000 | 1.74 (1.28, 2.37) | 0.000 | 1.97 (1.59, 2.45) | 0.000 |  |
| No education | 2.46 (1.66, 3.65) | 0.000 | 1.71 (1.06, 2.75) | 0.027 | 2.09 (1.55, 2.83) | 0.000 |  |
| **Husbands' education** |  |  |  |  |  |  |  |
| Higher | Ref |  | Ref |  | Ref |  | <0.001 |
| Secondary | 1.55 (1.20, 1.99) | 0.001 | 1.19 (0.93,1.52) | 0.173 | 1.33 (1.12, 1.59) | 0.001 |  |
| Primary | 1.91 (1.48, 2.49) | 0.000 | 1.46 (1.12,1.91) | 0.005 | 1.64 (1.36, 1.97) | 0.000 |  |
| No education | 1.98 (1.43, 2.74) | 0.000 | 1.89 (1.38, 2.59) | 0.000 | 1.86 (1.48, 2.32) | 0.000 |  |
| **Parity** |  |  |  |  |  |  |  |
| 1 | Ref |  | Ref |  | Ref |  | <0.001 |
| 2-3 | 1.78 (1.48, 2.14) | 0.000 | 1.12 (0.92, 1.37) | 0.260 | 1.44 (1.26, 1.65) | 0.000 |  |
| >3 | 2.74 (2.01, 3.74) | 0.000 | 1.61 (1.10, 2.38) | 0.015 | 2.17 (1.71, 2.76) | 0.000 |  |
| **Religion** |  |  |  |  |  |  |  |
| Others | Ref |  | Ref |  | Ref |  | <0.001 |
| Muslim | 1.40 (1.04, 1.90) | 0.027 | 1.82 (1.30, 2.54) | 0.000 | 1.57 (1.25, 1.98) | 0.000 |  |
| **Division** |  |  |  |  |  |  |  |
| Dhaka | Ref |  | Ref |  | Ref |  | <0.001 |
| Barisal | 0.82 (0.59, 1.14) | 0.243 | 1.12 (0.77, 1.61) | 0.548 | 0.96 (0.75, 1.23) | 0.757 |  |
| Chattogram | 1.13 (0.84, 1.51) | 0.431 | 0.90 (0.65, 1.24) | 0.521 | 1.00 (0.81, 1.25) | 0.976 |  |
| Khulna | 0.57 (0.41, 0.79) | 0.001 | 0.51(0.36, 0.72) | 0.000 | 0.55(0.44, 0.70) | 0.000 |  |
| Mymensingh | 1.08 (0.77, 1.52) | 0.650 | 1.11 (0.79, 1.57) | 0.540 | 1.10 (0.86, 1.40) | 0.436 |  |
| Rajshahi | 0.73 (0.53, 1.00) | 0.045 | 0.80 (0.58, 1.12) | 0.196 | 0.77(0.62, 0 .97) | 0.026 |  |
| Rangpur | 0.75 (0.53, 1.07) | 0.119 | 1.05 (0.75, 1.46) | 0.774 | 0.88 (0.69, 1.13) | 0.320 |  |
| Sylhet | 1.19 (0.85, 1.66) | 0.99 | 1.55 (0.84, 1.60)) | 0.379 | 1.18 (0.93, 1.50) | 0.178 |  |
| ***Enabling factors*** | | | | | | | |
| **Occupation** |  |  |  |  |  |  |  |
| Working | Ref |  | Ref |  | Ref |  | 0.002 |
| Not working | 0.78 (0.67, 0.92) | 0.003 | 1.02 (0.84,1.23) | 0.846 | 0.84 (0.75, 0.96) | 0.007 |  |
| **Husbands' occupation** |  |  |  |  |  |  |  |
| Working | Ref |  | Ref |  | Ref |  | 0.049 |
| Not working | 0.78 (0.37, 1.62) | 0.502 | 1.92 (1.06, 3.46) | 0.031 | 1.54 (0.95, 2.51) | 0.080 |  |
| **Wealth index** |  |  |  |  |  |  |  |
| Richest | Ref |  | Ref |  | Ref |  | <0.001 |
| Poorest | 4.55 (3.31, 6.26) | 0.000 | 2.11 (1.51, 2.95) | 0.000 | 3.20 (2.53, 4.03) | 0.000 |  |
| Poorer | 3.54 (2.62, 4.77) | 0.000 | 1.53 (1.13, 2.06) | 0.006 | 2.41 (1.95, 2.98) | 0.000 |  |
| Middle | 2.65 (2.01, 3.49) | 0.000 | 1.27 (0.95, 1.70) | 0.106 | 1.88 (1.53, 2.30) | 0.000 |  |
| Richer | 2.14 (1.66, 2.75) | 0.000 | 1.06 (0.78, 1.44) | 0.690 | 1.55 (1.28, 1.89) | 0.000 |  |
| **Media exposure^b^** |  |  |  |  |  |  |  |
| Any exposure | Ref |  | Ref |  | Ref |  | <0.001 |
| No exposure | 1.32 (1.09, 1.60) | 0.004 | 1.15 (0.97, 1.37) | 0.099 | 1.24 (1.10, 1.41) | 0.000 |  |
| **Accessing healthcare^c^** |  |  |  |  |  |  |  |
| Big problem | Ref |  | Ref |  | Ref |  | 0.420 |
| Not a big problem | 0.95 (0.81, 1.11) | 0.538 | 0.96 (0.81, 1.14) | 0.648 | 0.93 (0.83, 1.04) | 0.218 |  |
| **Residence** |  |  |  |  |  |  |  |
| Urban | Ref |  | Ref |  | Ref |  | <0.001 |
| Rural | 1.30 (1.06, 1.59) | 0.011 | 1.15 (0.92, 1.45) | 0.216 | 1.23 (1.06, 1.43) | 0.008 |  |
| ***Need factors*** | | | | | | | |
| **Terminated pregnancy** |  |  |  |  |  |  |  |
| No/never | Ref |  | Ref |  | Ref |  | <0.001 |
| Yes/ever | 0.72 (0.60, 0.86) | 0.000 | 0.85 (0.69, 1.05) | 0.130 | 0.76 (0.66, 0.87) | 0.000 |  |
| **Desired pregnancy** |  |  |  |  |  |  |  |
| Yes/then | Ref |  | Ref |  | Ref |  | 0.029 |
| No | 1.21 (1.00, 1.47) | 0.052 | 1.12 (0.91, 1.38) | 0.266 | 1.16 (1.01, 1.33) | 0.036 |  |
| ***Survey round*** | | | | | | | |
| BDHS 2017-18 |  |  |  |  | Ref |  | NA |
| BDHS 2022 |  |  |  |  | 1.01 (0.88, 1.15) | 0.929 |  |

^a^P-value obtained from joint Wald tests of the interaction between survey years and covariates; ^b^Media exposure: newspaper or television or radio; ^c^Problem to access healthcare: permission to go or monetary constrains or distance to health facilities; NA (not applicable)
