# Supplementary material for: Uncovering the Reasons Behind Maternal Care Dropout in Bangladesh: Cross-Sectional Study
Source: JMIR Public Health Surveill. 2026 Apr 1;12:e85875. doi: 10.2196/85875 (PMC13043009; doi:10.2196/85875)
Supplement: Multimedia Appendix 2 [file publichealth-v12-e85875-s002.docx]

**Supplementary Table 4: Fitness of regression models**

| **Covariates** | **Model 1: Dropout from ANC** | | **Model 2: Dropout from SBA delivery** | | **Model 3: Dropout from PNC** | | **Model 4: Dropout from all CoC components** | |
| --- | --- | --- | --- | --- | --- | --- | --- | --- |
|  | **Standard error of OR** | **VIF** | **Standard error of OR** | **VIF** | **Standard error of OR** | **VIF** | **Standard error of OR** | **VIF** |
| **Age at delivery** |  |  |  |  |  |  |  |  |
| < 19y | ref |  | ref |  | ref |  | ref |  |
| 19-30y | 0.08 | 2.24 | 0.07 | 2.24 | 0.07 | 2.24 | 0.07 | 2.24 |
| 31-49y | 0.10 | 2.65 | 0.07 | 2.65 | 0.08 | 2.65 | 0.08 | 2.65 |
| **Education level** |  |  |  |  |  |  |  |  |
| Higher | ref |  | ref |  | ref |  | ref |  |
| Secondary | 0.11 | 2.67 | 0.16 | 2.67 | 0.11 | 2.67 | 0.18 | 2.67 |
| Primary | 0.19 | 3.02 | 0.29 | 3.02 | 0.22 | 3.02 | 0.31 | 3.02 |
| No education | 0.46 | 1.75 | 0.42 | 1.75 | 0.32 | 1.75 | 0.46 | 1.75 |
| **Husbands' education** |  |  |  |  |  |  |  |  |
| Higher | ref |  | ref |  | ref |  | ref |  |
| Secondary | 0.13 | 2.33 | 0.16 | 2.33 | 0.12 | 2.33 | 0.19 | 2.33 |
| Primary | 0.19 | 2.83 | 0.21 | 2.83 | 0.15 | 2.83 | 0.24 | 2.83 |
| No education | 0.19 | 2.34 | 0.25 | 2.34 | 0.21 | 2.34 | 0.29 | 2.34 |
| **Occupation** |  |  |  |  |  |  |  |  |
| Working | ref |  | ref |  | ref |  | ref |  |
| Not working | 0.07 | 1.14 | 0.05 | 1.14 | 0.05 | 1.14 | 0.06 | 1.14 |
| **Husbands' occupation** |  |  |  |  |  |  |  |  |
| Working | ref |  | ref |  | ref |  | ref |  |
| Not working | 0.29 | 1.01 | 0.20 | 1.01 | 0.38 | 1.01 | 0.32 | 1.01 |
| **Parity** |  |  |  |  |  |  |  |  |
| 1 | ref |  | ref |  | ref |  | ref |  |
| 2-3 | 0.08 | 1.65 | 0.13 | 1.65 | 0.10 | 1.65 | 0.13 | 1.65 |
| >3 | 0.22 | 1.91 | 0.34 | 1.91 | 0.27 | 1.91 | 0.35 | 1.91 |
| **Terminated pregnancy** |  |  |  |  |  |  |  |  |
| No/never | ref |  | ref |  | ref |  | ref |  |
| Yes/ever | 0.05 | 1.04 | 0.05 | 1.04 | 0.05 | 1.04 | 0.06 | 1.04 |
| **Desired pregnancy** |  |  |  |  |  |  |  |  |
| Yes/then | ref |  | ref |  | ref |  | ref |  |
| No | 0.08 | 1.10 | 0.09 | 1.10 | 0.08 | 1.10 | 0.10 | 1.10 |
| **Wealth index** |  |  |  |  |  |  |  |  |
| Richest | ref |  | ref |  | ref |  | ref |  |
| Poorest | 0.30 | 3.00 | 0.54 | 3.00 | 0.38 | 3.00 | 0.60 | 3.00 |
| Poorer | 0.26 | 2.46 | 0.40 | 2.46 | 0.26 | 2.46 | 0.46 | 2.46 |
| Middle | 0.20 | 2.02 | 0.27 | 2.02 | 0.19 | 2.02 | 0.32 | 2.02 |
| Richer | 0.15 | 1.82 | 0.21 | 1.82 | 0.15 | 1.82 | 0.28 | 1.82 |
| **Media exposure^α^** |  |  |  |  |  |  |  |  |
| Any exposure | ref |  | ref |  | ref |  | ref |  |
| No exposure | 0.09 | 1.32 | 0.09 | 1.32 | 0.08 | 1.32 | 0.11 | 1.32 |
| **Accessing healthcare^β^** |  |  |  |  |  |  |  |  |
| Big problem | ref |  | ref |  | ref |  | ref |  |
| Not a big problem | 0.05 | 1.08 | 0.06 | 1.08 | 0.05 | 1.08 | 0.06 | 1.08 |
| **Religion** |  |  |  |  |  |  |  |  |
| Others | ref |  | ref |  |  |  |  |  |
| Muslim | 0.18 | 1.04 | 0.23 | 1.04 | 0.19 | 1.04 | 0.26 | 1.04 |
| **Residence** |  |  |  |  |  |  |  |  |
| Urban | ref |  | ref |  | ref |  | ref |  |
| Rural | 0.11 | 1.25 | 0.11 | 1.25 | 0.10 | 1.25 | 0.12 | 1.25 |
| **Division** |  |  |  |  |  |  |  |  |
| Dhaka | ref |  | ref |  | ref |  | ref |  |
| Barisal | 0.16 | 1.63 | 0.13 | 1.63 | 0.12 | 1.63 | 0.16 | 1.63 |
| Chattogram | 0.13 | 1.82 | 0.14 | 1.82 | 0.11 | 1.82 | 0.15 | 1.82 |
| Khulna | 0.10 | 1.58 | 0.07 | 1.58 | 0.07 | 1.58 | 0.07 | 1.58 |
| Mymensingh | 0.09 | 1.71 | 0.15 | 1.71 | 0.14 | 1.71 | 0.12 | 1.71 |
| Rajshahi | 0.13 | 1.59 | 0.10 | 1.59 | 0.09 | 1.59 | 0.10 | 1.59 |
| Rangpur | 0.08 | 1.72 | 0.12 | 1.72 | 0.11 | 1.72 | 0.09 | 1.72 |
| Sylhet | 0.15 | 1.72 | 0.16 | 1.72 | 0.14 | 1.72 | 0.19 | 1.72 |
| **Survey round** |  |  |  |  |  |  |  |  |
| BDHS 2017-18 | ref |  | ref |  | ref |  | ref |  |
| BDHS 2022 | 0.09 | 1.08 | 0.03 | 1.08 | 0.07 | 1.08 | 0.04 | 1.08 |

^α^Media exposure: newspaper or television or radio; ^β^Problem to access healthcare: permission to go or monetary constrains or distance to health facilities
