# Supplementary material for: Uncovering the Reasons Behind Maternal Care Dropout in Bangladesh: Cross-Sectional Study
Source: JMIR Public Health Surveill. 2026 Apr 1;12:e85875. doi: 10.2196/85875 (PMC13043009; doi:10.2196/85875)
Supplement: Checklist 1 [file publichealth-v12-e85875-s003.docx]

**STROBE Checklist: Strengthening the Reporting of Observational Studies in Epidemiology**

Title and Abstract

| Item | Recommendation | Reported | Comments |
| --- | --- | --- | --- |
| 1 | Indicate study design with a commonly used term in the title or abstract | Yes | ﻿Title |
| 2 | Provide an informative and balanced abstract summarizing objectives, methods, key results, and conclusions | Yes | ﻿Abstract |

Introduction

| Item | Recommendation | Reported? | Comments |
| --- | --- | --- | --- |
| 3a | Explain the scientific background and rationale for the investigation | Yes | Paragraph 6 |
| 3b | Clearly state specific objectives or hypotheses | Yes | Paragraph 6 |

Methods

Study Design and Setting

| Item | Recommendation | Reported? | Comments |
| --- | --- | --- | --- |
| 4a | Present key elements of study design early in the paper | Yes | Paragraph 1 of study design, data sources, and sampling |
| 4b | Describe the setting, locations, and relevant dates, including periods of recruitment, exposure, follow-up, and data collection | Yes | Paragraph 2 of study design, data sources, and sampling and figure 1 |

Participants

| Item | Recommendation | Reported? | Comments |
| --- | --- | --- | --- |
| 5a | Cohort study: Give the eligibility criteria and sources and methods of selection of participants; describe methods of follow-up |  |  |
| 5a | Case-control study: Give eligibility criteria and sources and methods of case and control selection |  |  |
| 5a | Cross-sectional study: Give eligibility criteria and sources and methods of selection of participants | Yes | Paragraph 2 of study design, data sources, and sampling and figure 1 |
| 5b | Cohort study: For matched studies, give matching criteria and number of exposed and unexposed |  |  |
| 5b | Case-control study: For matched studies, give matching criteria and the number of cases and controls |  |  |

Variables

| Item | Recommendation | Reported? | Comments |
| --- | --- | --- | --- |
| 6a | Clearly define outcomes, exposures, predictors, potential confounders, and effect modifiers | Yes | outcome variable and covariates |
| 6b | Give sources of data and details of methods of assessment (measurement) for each variable used in the study | Yes | ﻿Statistical analysis |

Data Sources/Measurement

| Item | Recommendation | Reported? | Comments |
| --- | --- | --- | --- |
| 7 | Describe comparability of assessment methods if there is more than one group | Yes | ﻿Statistical analysis |

Bias

| Item | Recommendation | Reported? | Comments |
| --- | --- | --- | --- |
| 8 | Describe any efforts to address potential sources of bias | Yes | ﻿Statistical analysis |

Study Size

| Item | Recommendation | Reported? | Comments |
| --- | --- | --- | --- |
| 9 | Explain how the study size was arrived at | Yes | Paragraph 1 and 2 of study design, data sources, and sampling and Figure 1 |

Quantitative Variables

| Item | Recommendation | Reported? | Comments |
| --- | --- | --- | --- |
| 10 | Explain how quantitative variables were handled in the analyses; if applicable, describe which groupings were chosen and why | Yes | ﻿Statistical analysis |

Statistical Methods

| Item | Recommendation | Reported? | Comments |
| --- | --- | --- | --- |
| 11a | Describe all statistical methods, including those used to control for confounding | Yes | ﻿Statistical analysis |
| 11b | Describe any methods used to examine subgroups and interactions | ﻿ N/A |  |
| 11c | Explain how missing data were addressed | Yes | ﻿ Statistical analysis and figure 1 |
| 11d | Cohort study: If applicable, explain how loss to follow-up was addressed |  |  |
| 11e | Case-control study: If applicable, explain how matching of cases and controls was accounted for in the analysis |  |  |
| 11f | Cross-sectional study: Describe analytical techniques used to account for sampling strategy, particularly stratification and clustering | Yes | ﻿Study design, data source and  sampling |

Results

Participants

| Item | Recommendation | Reported? | Comments |
| --- | --- | --- | --- |
| 12a | Report numbers of individuals at each stage of study; diagram if helpful | Yes | Figure 1 |
| 12b | Report reasons for nonparticipation at each stage | Yes | Figure 1 |
| 12c | Consider use of a flow diagram | Yes | Figure 1 |

Descriptive Data

| Item | Recommendation | Reported? | Comments |
| --- | --- | --- | --- |
| 13a | Give characteristics of study participants (demographic, clinical, social) and information on exposures and potential confounders | Yes | Table 1 |
| 13b | Indicate number of participants with missing data for each variable of interest | Yes | Figure 1 |
| 13c | Cohort study: Summarize follow-up time (e.g., average and total amount) |  |  |

Outcome Data

| Item | Recommendation | Reported? | Comments |
| --- | --- | --- | --- |
| 14a | Report numbers of outcome events or summary measures over time | Yes | Paragraph 1 of result and figure 3 |
| 14b | Cohort study: Report the unadjusted estimate and its precision (confidence interval); adjust for confounders |  |  |
| 14b | Case-control study: Report both unadjusted and adjusted odds ratios and their precision and confounders adjusted for |  |  |
| 14b | Cross-sectional study: Report prevalence ratios and their precision; adjust for confounders if applicable | Yes | Paragraph 1 of result, figure 3, table 2 |
| 14c | Report category of exposure if applicable | Yes | Table 1 |
| 14d | If relevant, consider translating estimates of relative risk into absolute differences for a more intuitive understanding | N/A |  |

Other Analyses

| Item | Recommendation | Reported? | Comments |
| --- | --- | --- | --- |
| 15 | Report other analyses done—e.g., analyses of subgroups and interactions, and sensitivity analyses | N/A |  |

Discussion

| Item | Recommendation | Reported? | Comments |
| --- | --- | --- | --- |
| 16 | Summarize key findings with reference to study objectives | Yes | ﻿Paragraph 1 |
| 17 | Discuss limitations of the study, taking into account sources of potential bias or imprecision | Yes | last paragraph |
| 18a | Discuss interpretation of findings considering direction and magnitude of associations, dose-response relations, plausibility, comparison with other studies, and alternative explanations | Yes | Paragraph 7, 8,9 |
| 18b | Describe how findings may be generalizable or applicable to other populations | Yes | Conclusion |

Other Information

| Item | Recommendation | Reported? | Comments |
| --- | --- | --- | --- |
| 19 | Give the source of funding and the role of the funders for the present study and, if applicable, for the original study on which the present article is based | Yes | Funding |
| 20 | Declare author conflicts of interest | Yes | Competing interest |
